# Ethnopharmacological Survey and Comparative Study of the Healing Activity of Moroccan Thyme Honey and Its Mixture with Selected Essential Oils on Two Types of Wounds on Albino Rabbits

**DOI:** 10.3390/foods11010028

**Published:** 2021-12-23

**Authors:** Mouna Mekkaoui, Hamza Assaggaf, Ahmed Qasem, Adel El-Shemi, Emad M. Abdallah, El Houcine Bouidida, Hanae Naceiri Mrabti, Yahya Cherrah, Katim Alaoui

**Affiliations:** 1 Pharmacodynamics Research Team ERP, Laboratory of Pharmacology and Toxicology, Faculty of Medicine and Pharmacy, University Mohammed V in Rabat, Rabat BP 6203, Morocco; alaouikma@yahoo.fr; 2 Laboratory Medicine Department, Faculty of Applied Medical Sciences, Umm Al-Qura University, Makkah 21955, Saudi Arabia; hmsaggaf@uqu.edu.sa (H.A.); Aaqasem@uqu.edu.sa (A.Q.); aghsemi@uqu.edu.sa (A.E.-S.); 3 Department of Science Laboratories, College of Science and Arts, Qassim University, Ar Rass 51921, Saudi Arabia; emad100sdl@yahoo.com; 4 National Laboratory of Drugs Controlled, Rabat BP 6203, Morocco; bouididae@yahoo.fr; 5 Laboratory of Pharmacology and Toxicology, Bio Pharmaceutical and Toxicological Analysis Research Team, Faculty of Medicine and Pharmacy, University Mohammed V in Rabat, Rabat BP 6203, Morocco; naceiri.mrabti.hanae@gmail.com; 6 Biopharmaceutical and Toxicological Analysis Research Team, Laboratory of Pharmacology and Toxicology, Faculty of Medicine and Pharmacy, University Mohammed V in Rabat, Rabat BP 6203, Morocco; cherrahy@yahoo.fr

**Keywords:** honey, essential oils, synergy, survey, toxicity, antimicrobial, wound healing, in vivo, rabbits

## Abstract

Wound healing consists of several continuous phases involving various cells and chemical intermediates. As a rich source of nutrition elements, honey has proved to have potential benefits in the treatment of various diseases. The present study was designed to investigate the healing effect of a honey mixture with selected essential oils on chemical and thermal wound models in rabbits. Dressing mixtures of *Thymus vulgaris* honey with three essential oils (*Origanum vulgare, Rosmarinus officinalis,* and *Thymus vulgaris*) were prepared and applied daily in the treatment groups. These essential oils were rich in phytochemicals and had significant antibacterial activity against four selected ATCC bacterial strains. Madecasol ointment was used as a standard control. The healing effect of the mixtures was evaluated by measuring wound surface area and comparing healing time. The results showed that the healing rate in the treatment groups was significantly higher than that of the untreated group and standard group. The best healing effect for burns was seen in the mixture of honey and *Thymus vulgaris* essential oil, which had wound closure rates of 85.21% and 82.14% in thermal- and chemical-induced burns, respectively, and showed the shortest healing time (14 days) in comparison to other groups. Therefore, it can be concluded that honey mixtures have significant beneficial effects on skin wound healing and, thus, they may be used as a healing agent in different types of wounds in humans after specific clinical trials.

## 1. Introduction

The skin is an exterior organ that covers the body and provides many vital functions, including organ protection, percutaneous absorption, body shape maintenance, fluid conservation, temperature control, and sensory and disease control [1,2]. Honey is a rich source of essential minerals and bioactive molecules with various therapeutic features [3]. For thousands of years, honey has been used in topical wound dressing [4]. The unique antibacterial, anti-inflammatory, and antioxidant properties of honey contributes to wound healing, especially in ulcers and burns [5,6]. A large number of publications in the twenty-first century evidenced the properties of honey in wound management and other medical applications of honey. In Morocco, recent studies have revealed various uses of Moroccan honey in biological applications. Imtara et al. [7,8] evaluated the antioxidant and wound healing effects of Tulkarm honey and *Thymus vulgaris* honey. The study of El-Haskoury et al. [9] covered the antioxidant activity of carob honey. Elamine et al. [10] reviewed the physicochemical characteristics and antioxidant activities of Moroccan Zantaz honey, and El-Guendouz et al. [11] studied the antioxidant and diuretic activities of *Capparis Spinosa* honey and propolis.

Plants also are used for treating wounds and burns by many traditional practitioners across the world. Ointments extracted from medicinal plants have been used as healing agents due to their wide variety of different constituents, such as alkaloids, essential oils, flavonoids, tannins, terpenoids, saponins, fatty acids, and phenolic compounds, which are all capable of enhancing the healing process of the burn [12]. The presence of bioactive constituents in plants has urged researchers to screen medicinal plants to determine potential wound healing activities and isolate chemical entities associated with wound healing [13]. The position of Morocco between two seas and a vast desert, crossed by four mountain chains, results in a complete range of Mediterranean bioclimates. This environmental diversity provides a habitat for a rich and varied flora, with about 4500 species, including 600 plants, with medicinal and aromatic properties [14,15]. Several ethnobotanical surveys among Moroccan herbalists have shown various plants to treat wounds. In the recent survey conducted in Agadir city by El-Ghazouani et al. eight plants were reported by herbalists to treat wounds [16]. In the area of Rabat, Salhi et al. recorded 36 species used by herbalists in the treatments of skin burns [17], and in oriental Morocco, Fakchich et al. reported four plants [18]. Despite the variety of plants highlighted in the surveys above, only a few studies on the healing activity of plants have been conducted in Morocco. Nejari et al. [19] and Jawhari et al. [20] studied the wound healing properties of Telephium imperati L. and Anacyclus pyrethrum L, respectively.

Rare are the studies on combining honey with essential oils or plant extracts. Imtara et al. [21] researched the antibacterial effect of honey combined with *Origanum vulgare* L. essential oil. On the other hand, Boukraâ et al. [22] studied the synergistic effect of monofloral honey and essential oils against *Pseudomonas aeruginosa*, and El Ouardy et al. [23] tested the antibacterial activity of the mixture honey/*Mentha pulegium* essential oil, while Belmehdi et al. [24] investigated the antibacterial activity of the combination of propolis extracts with essential oils and antibiotics. To the best of our knowledge, none of the studies conducted have investigated the synergistic effect between honey and essential oils for treating wounds. As a result, the primary goal of this study is to assess the wound healing capacity and antibacterial potential of oregano, rosemary, and thyme essential oils mixed with Moroccan thyme honey on thermal and chemical wound models in rats.

## 2. Material and Methzods

### 2.1. Study Area and Survey

**Figure 1:**
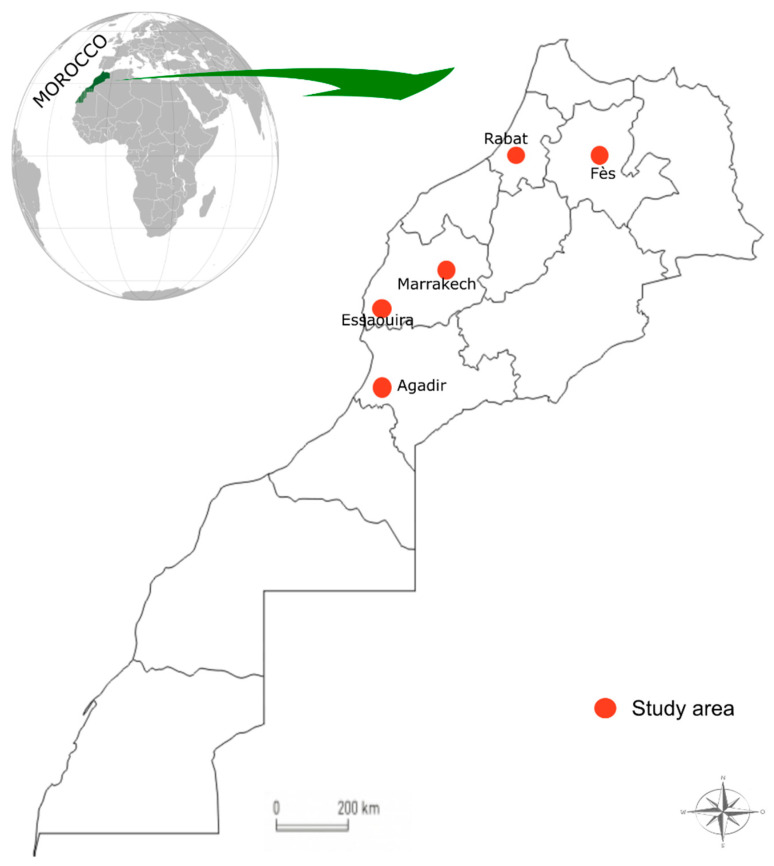
Study area.

An ethnobotanical survey was conducted between June 2017 and October 2018 in five cities of Morocco: Agadir, Essaouira, Marrakech, Meknes, and Rabat (Figure 1). A total of 173 herbalists and 147 beekeepers were consulted.

The survey was carried out with the help of two questionnaires, written in French and Arabic. In this survey, the first questionnaire (Table 1) addressed the sociodemographic characteristics of herbalists (age, gender, years of experience, city, area, level of education, and sources of ethno-medicinal knowledge) and details of plants used in traditional medicine for the treatment of wounds, their route of administration, the parts used, and preparation methods.

The second questionnaire (Table 2) addressed the sociodemographic profiles of beekeepers (age, sex, years of experience, city, area, level of education, and sources of ethnomedicinal knowledge) and information about the apiary (size of the apiary, method of beekeeping, method of extraction, marketing circuit adapted). Moreover, the questionnaire included all information about honey (most sold type(s) of honey, type(s) of honey used for wound healing, and their method of administration).

The collected data were transferred to a database and analyzed by statistical processing software IBM(SPSS), version 26, and Excel 2016.

### 2.2. Honey Sample Collection

Three samples of *Thymus vulgaris* honey were purchased from local professional beekeepers in the region of Essaouira, precisely from the village of Tamanar. Harvesting, extraction, and packaging were carried out using a traditional method. The samples were stored in sealed plastic jars, followed by labeling and dating, and kept at room temperature ± 29 °C until the end of the analysis.

### 2.3. Physicochemical Properties of Thymus Vulgaris Honey

Water content (moisture) was determined by an Erma Refractometer reading at 20 °C using the Wedmore table, and the results were expressed as percentages. pH was measured by a pH-meter (ProLab3000), using a solution containing 10 g of honey dissolved in 75 mL carbon dioxide-free water.

The electrical conductivity was obtained by the method of Vorwohl (1964), where the electrical resistance at 20 °C of a 20% honey solution is measured with a Crison Basic 30 conductimeter. The results were expressed in millisiemens per centimeter (mS/cm).

The free lactonic and total acidities were determined by a titrimetric method using a solution containing 10 g honey in 75 mL of CO_2_-free distilled water. The titration was carried out using 0.1 M NaOH and stopped at pH 8.5. The lactone acidity was calculated as 10 times the volume of 0.1 M NaOH used for neutralization of 10 g of honey and expressed in milliequivalents of acid per kg of honey (meq/kg). Total acidity results were obtained by adding free plus lactone acidities [25].

A honey refractometer determined sugar content with a direct reading display, and the results were expressed as brix degrees. Hydroxymethylfurfural (HMF) was determined by clarifying samples with Carrez reagents (I and II). The absorbance was measured at 284 nm and 336 nm in a 1 cm quartz cuvette in a spectrophotometer (Milton Roy UV–vis Spectronic 3000 Array). Ash percentage was determined by calcinating the sample in a muffle furnace at 500 °C until a constant mass was attained, and the results were expressed as percentages (Horwitz 2010).

### 2.4. Essential Oils Selection and Analysis

The selection of essential oils used in this study was based on the results of the ethnopharmacological survey combined with bibliographical research using electronic databases, such as PubMed, Scopus, and ScienceDirect. The search was conducted with “essential oils” and “wound healing” as the primary keywords. From this search, only results including an animal model were considered. In a second step, “honey mixture” and “dressing” were added to the exact previous keywords; works devoted to combining EO and honey aimed at wound healing were selected.

Three essential oils with high wound healing properties (*Origanum vulgare*, *Rosmarinus officinalis*, and *Thymus vulgaris* essential oils) were selected for this study. The three samples were provided by the company EDEPAM, a company of distillation and exploitation of aromatic and medicinal plants in Kenitra.

The chemical characterization of the three essential oils was analyzed and identified by a gas chromatography/mass spectrometry (GC/MS) approach, using a Hewlett-Packard (HP6890) GC instrument coupled with a HP5973 MS and equipped with a 5% phenylmethyl silicone HP-5MS capillary column (30 m × 0.25 mm × film thickness 0.25 μm). Briefly, the column temperature was started at 50 °C for 5 min and then increased to 200 °C with a 4 °C/min rate. The carrier gas was helium with a 1.5 mL/min flow rate and split mode (flow: 112 mL/min, ratio: 1/74.7). The temperature of the injector and detector was 250 °C, and the hold time was 48 min. The machine was led by a computer system type ″HP ChemStation″, managing the functioning of the machine and allowing us to follow the evolution of chromatographic analyses. Diluted samples (1/20 in methanol) of 1 μL were injected manually. The MS operating conditions were: 70 eV ionization voltage, 230 °C ion source temperature, and 35–450 (m/z) scanning range. The qualitative analyses of the different compounds were based on the percent area of each peak of the sample compounds and were confirmed by reference to their MS identities (Library of NIST/EPA/NIH MASS SPECTRAL LIBRARY Version 2.0, build 1 July 2002) [26]. 

### 2.5. Antibacterial Activity

#### 2.5.1. Preparation of Bacterial Strains

The examined bacterial species, including *Escherichia coli* ATCC 25922, *Salmonella Typhimurium* ATCC 700408, *Staphylococcus aureus* ATCC 29213, and *Listeria monocytogenes* ATCC 13932, were prepared by inoculating a loopful from the frozen stock (−20 °C) in Mueller-Hinton agar (Biokar, Beauvais, France) and incubated at 37 °C for 24 h.

#### 2.5.2. Disc Diffusion Assay

The preliminary evaluation of the antibacterial capacity of essential oils was performed by a disc diffusion assay according to the protocol described previously [27], with few modifications. Firstly, the studied essential oils were mixed with dimethyl sulfoxide (DMSO) at a concentration of 5% to facilitate their diffusion in the culture medium. At the same time, a bacterial suspension of 0.5 McFarland (10^8^ CFU/mL) representing each examined bacterium was prepared in physiological water (0.9% NaCl) and inoculated by swabbing on plates containing Mueller-Hinton agar (Biokar, Beauvais, France). Then, 10 µL of each essential oil was dropped on 6 mm diameter sterile paper discs. At the same time, a disc containing 10 µL of DMSO at a concentration of 5% was used as a negative control, and chloramphenicol (30 µg) was used as a reference test. Afterward, all the plates were incubated at 37 °C for 24 h. After incubation, the inhibition diameter was measured in millimeters (disk included) and expressed as mean ± standard deviation of three replicates.

#### 2.5.3. Determination of MIC and MBC

The minimum inhibitory concentration (MIC) of each examined essential oil was determined by microbroth dilution in 96-well microplates [28]. Decreasing concentrations of each essential oil were prepared in DMSO by using the serial two-fold dilution method in each microplate row. Then, 20 μL of 0.5 McFarland bacterial suspension and 160 μL of Mueller-Hinton broth (MHB, Biokar, Beauvais, France) were added, and the microplates were incubated at 37 °C for 24 h. Afterward, the bacterial growth was checked by adding 40 μL of 2,3,5-triphenyltetrazolium chloride (TTC) (Sigma-Aldrich, Schaffhausen, Switzerland) with a 0.2 g/mL concentration, followed by incubation for 30 min at 37 °C. The TTC stains the bacteria red, indicating the wells showing bacterial growth [29]. The MIC was considered the MIC, because the microplate wells contained a lower concentration of essential oils and did not show visible bacterial growth. However, minimum bactericide concentration (MBC) was determined by sub-culturing 50 μL from the microplate well that did not present bacterial growth on Mueller-Hinton agar (Biokar, Beauvais, France). The plates were incubated at 37 °C for 24 h. The lower concentration that did not present any media growth was considered the MBC. This study used chloramphenicol (30 μg/disc) (Sigma-Aldrich, Schaffhausen, Switzerland) as a reference test.

### 2.6. Honey and Essential Oil Mixtures

The mixtures of honey/essential oil were prepared according to the acute dermal irritation assay and the bibliographical research. For each preparation, 0.5% of essential oil was mixed in 100 g of honey while stirring for 5 min, using a wooden spoon to avoid the interference of metal molecules. The three mixtures were to serve for external use only.

### 2.7. Animals

42 Wistar rats (180–200 g, aged 3–5 months) were used to study acute dermal toxicity, and 36 rabbits for the wound healing activity test. All animals were obtained from the Animal Center of the National Laboratory to Control Medicines at the Directorate of Medicines and Pharmacy in Rabat.

The animals were housed in individual cages in temperature-controlled (23 ± 2 °C) and artificially lighted rooms on a 12 h light/12 h dark cycle with free access to water and a standard diet.

### 2.8. Ethics Approval

The studies were carried out following the guidelines in the “Guide for the Care and Use of Laboratory Animals” prepared by the National Academy of Sciences and published by the National Institutes of Health. Ethical approval was obtained from Mohammed V University in Rabat.

### 2.9. Acute Dermal Irritation Assay for Essential Oils

A total of 42 rats were used to test the acute dermal irritation for the three essential oils, and they were divided into seven groups (*n* = 6). The first group (I) served as control; groups II and III received, respectively, 0.5% and 5% of *Origanum vulgare* essential oil (OEO); groups IV and V received 0.5% and 5% of *Thymus vulgaris* essential oil (TEO); and groups VI and VII, 0.5% and 5% of *Rosmarinus officinalis* essential oil (REO). Within 24 h before the test, the fur was removed from the dorsal area of the trunk of each rat, being careful to avoid abrading the skin. The application and observation times were identified according to the method described by Craig et al. [30], where the essential oil was applied to a small area of skin (5 cm × 5 cm) and covered with a gauze patch. Access by the animal to the patch and resultant ingestion/inhalation was prevented. At the end of 4 h exposure period, the residual extract was removed, and each site was examined for erythema and edema. 

The scoring was determined by the method of Draize [31], where 0 is for no erythema and erythema scores are 1 for very slight, 2 for well defined, 3 for moderate to severe, and 4 for severe to eschar formation. Edema was scored similarly, with 0 indicating none, 1 very slight, 2 slight, 3 moderate, and 4 severe. A score for each animal was determined using the immediate, 24 h, 48 h, 72 h, and 5 day observations.

### 2.10. Wound Healing Study

Two experimental trials were used to evaluate the healing activity of thyme honey and mixtures; the first protocol was for thermal burns and the second for chemical burns. For both protocols, 36 rabbits were used and divided into six groups (*n* = 6).

Thymus vulgaris honey was used to treat group I (TH). Group II received a mixture of Thymus vulgaris honey and *Origanum vulgare* essential oil (TH-O). Group III was given a combination of Thymus vulgaris honey and Thymus vulgaris essential oil (TH-T). Group IV was given a mixture of Thymus vulgaris honey and *Rosmarinus officinalis* essential oil (TH-R); group V was given the conventional medicine (Madecassol^®^); and group VI was given simply Vaseline as therapy. The animals were treated every 24 h. Honey and mixes were applied topically to cover the wound region.

#### 2.10.1. Induction of Wound

Wounds were induced in the same manner as reported by Imtara et al. [32], except for one modification in the diameter of the metal rod. All groups were subjected to chemical and thermal wounding, and wounds were induced on either side of the rabbits’ dorsal skin simultaneously. The hair on the dorsal skin of the rabbits was shaved manually using an electric razor and then marked with ink. The animals were separated for 24 h to see any recurrence of skin injury induced by shaving. The thermal injury was used to activate the thermally induced wound, and direct heat was administered using a hot stainless-steel metal rod with a 2.2 cm diameter and kept at 80 °C. As detailed by Al-Saeed et al. [33] and Abu-Zinadah et al. [34], the chemically generated wound was influenced by distributing a few drops of concentrated HCl (35% *v*/*v*) onto the shaved skin of rabbits. The wound area’s diameter was assessed at 1 day, 3 day, 5 day, 1 week, and 2 week intervals.

#### 2.10.2. Wound Closure

Every two days, the size of the lesions was measured on transparency paper. The wound surface areas were then measured using a design and drawing software tool (AutoCAD 2019). Wound contraction was computed as a reduction in the initial wound size, using the equation employed by Bouassida et al. [35] under comparable conditions, which is:

$$Wound\;closure\;\left(\%\right)=\left[\frac{\left(A_{0}-A_{d}\right)}{A_{0}}\right]\times 100$$

where *A*_0_ and *A_d_* are initial wound area and wound area on day (d), respectively.

### 2.11. Statistical Analysis

For all measures, the results were expressed as the mean standard deviation of triplicate analyses. The analysis of variance (ANOVA) for comparison of sample means was used to analyze variations in observed parameters among the samples. Data were statistically analyzed using the SPSS softwar package, version 26.

## 3. Results and Discussion 

### 3.1. A Survey on Honey Types Used in Treatment

#### 3.1.1. Sociodemographic Characteristics of Beekeepers

The sociodemographic characteristics are essential criteria in ethnopharmacological studies. In our study, 147 beekeepers participated in the survey in five Moroccan cities, Rabat, Fes, Essaouira, Agadir, and Marrakech. The average age of the Moroccan beekeeper is 47 years old, with the most dominant age range being between 45 and 58 years old. It is important to note that most beekeepers interviewed (85.71%) are men, and 14.29% are women, which justifies the fact that this field in Morocco remains a very masculine domain (Table 3). Moroccan culture can explain this result, which does not encourage women to do this type of work. To the best of our knowledge, no ethnopharmacological investigation of beekeepers has been carried out in Morocco to use honey in the treatment of wounds. In addition, the results of the present study showed that half of the beekeepers have secondary education (48.3%), 32.65% have primary education or have attended Koranic schools, 16.33% are university students, and only 6.12% are illiterate. These findings showed that most information about medicinal knowledge is conserved and transmitted by people who do not have an excellent intellectual level. Therefore, this way of transmission can affect the fidelity of the information transmitted.

#### 3.1.2. The Diversity of Honey Used for Wound Healing

Honey is one of the most complex foodstuffs found in nature and, indeed, the only sweetening agent that humans can use without processing [36]. Since ancient times, it has been regarded as a healthy food for its wide pharmacological activities, including antibacterial, antioxidant, anti-inflammatory, immunomodulation, and antitumor [37,38,39,40,41,42].

Morocco has a long tradition of beekeeping, with a total annual honey production estimated at 3500 tons, of which eucalyptus, thyme, euphorbia, citrus, and carob kinds of honey account for the largest share; however, thyme and euphorbia kinds of honey are the most popular with consumers [36].

**Figure 2:**
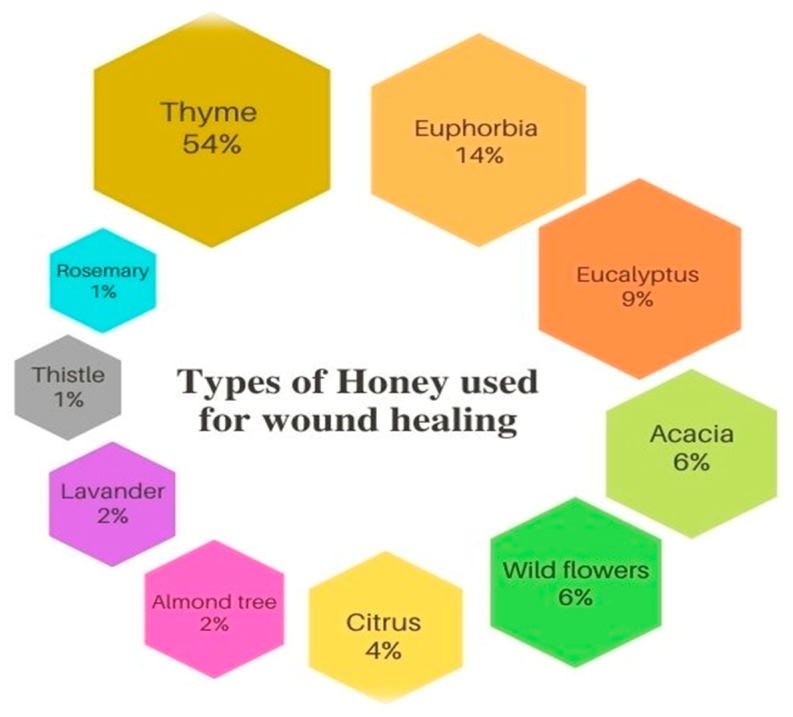
Honey used for wound healing.

In our study, 10 varieties of honey were recorded as wound healing agents (Figure 2). The most common varieties of honey used to treat wounds are *Thymus vulgaris* honey (54%), followed by *Euphorbia resinifera* honey (14%), and *Eucalyptus globulus* honey (9%).

This result could be explained by the recent study of Imtara et al. [8], where the former authors pointed out a high content of phenols, flavonoids, and flavanols. HPLC analysis showed for the first time that Moroccan *Thymus vulgaris* honey contains epicatechin gallate, and this component effectively boosts the wound healing process in several studies [43,44,45].

### 3.2. Survey on Wound Healing Plants

Morocco has an important floristic diversity of which medicinal plants constitute a remarkable percentage. Plants have been used since antiquity as a primary renewable source of drugs to treat various diseases and disorders [46]. This fact could be attributed to their broad biological and medicinal activities. Therefore, medicinal herbs play an important role in developing potent therapeutic agents [47].

Our study summarizes the results obtained on medicinal plants used for wound healing in Table 4. The table lists scientific names of species, botanical families, local names, plant parts used, preparation methods, mode of application, type of wound, and frequency of citations.

As listed in Table 4, 29 species belonging to 16 plant families have been identified as wound healing agents used to treat skin afflictions such as sores, bites, burns, and lacerations. These results confirm the diversity of medicinal plants used in these regions. This diversity is explained by the massive Moroccan floristic richness [48,49,50]. Among the 16 botanical families recorded in the study areas, the Lamiaceae family contributed the highest number of plants, with 10 species, followed by the Asteraceae family, with three species, and the Euphorbiaceae and Pinaceae families, with two plants each. In contrast, the other families have only one species. The predominance of these families has been observed in several ethnopharmacological surveys from Morocco [51,52,53,54,55,56]. The majority of these families are utilized, due to the richness of the Moroccan flora [17]. In addition, the Lamiaceae family is characterized by essential oils with an essential role in therapies because of their exciting chemical composition and broad spectrum of biological activities [51]. Leaves were the most frequently used parts (55%), followed by the stem (17%), seeds (11%), fruits (8%), barks, resin, and bulbs (3%). The use of leaves to treat dermatological wounds and diseases could be explained by the availability and richness of therapeutic substances [17].

Regarding the most quoted plants, it is clear that *Thymus vulgaris*, *Origanum vulgare*, and *Rosmarinus officinalis* are the most used to heal wounds. Previous studies have experimentally demonstrated the wound healing activity of these species; *Rosmarinus officinalis* has shown a high contraction rate in excision wounds [57,58]. The essential oil of *Origanum vulgare* has led to a significant reduction in the areas of wounds treated and showed an excellent capacity for tissue remodeling, particularly re-epithelialization [59]. *Thymus vulgaris* was recommended as a natural agent for wounds by protecting the wound site from infection, inhibiting inflammatory cells, and increasing the formation of connective tissue in repaired tissues [60,61,62].

### 3.3. Physicochemical Properties of Thyme Honey

The results of physicochemical parameters are shown in Table 5.

Data showed that all of the samples’ analytical parameters conformed with the limits set in the standard for honey [63]. The moisture content of the three honey samples ranged from 16.93% to 17.5%, and all percentages were within the international limit (≤21%). Honey moisture content depends on several factors, such as degree of maturity reached, yielding season, and ecological factors [64].

Honey is naturally acidic; this acidity may be due to organic acids that give it its flavor and allow it to resist microbial spoilage. The pH is also important, because it affects the texture, stability, and shelf life [65]. The samples varied in pH from 4.11 to 4.67, showing almost the same range as recorded by Laredj et al. [39] and Elimam et al. [66]. In the same way, electrical conductivity ranges obtained by Boussaid et al. [67] (0.39–0.89 mS/cm), Lokossou et al. [68] (0.37–1.43 mS/cm), and Guler et al. [69] (0.250–0.90 mS/cm) in Tunisian, Beninese, and Turkish honey had more or less identical results to those found in our thyme honey samples (0.73–0.87 mS/cm).

Acidity in honey varies due to floral origins and harvesting seasons [70]. The acidity of thyme honey samples (33.9–39.7 meq/kg) was within the international limits. Another parameter used for the determination of the botanical origin is ash content. Our samples’ ash contents (0.17– 0.4%) were within the Codex Alimentarius Standards (≤0.6 g/kg).

### 3.4. Antimicrobial Potential of the Essential Oils

The antibacterial testing of the essential oils was carried out to evaluate the possible prevention and disinfection power of these essential oils against bacterial infection in injured tissue during the stimulation of wound healing processes.

Wound healing is a dynamic and complicated biological process that includes phases of inflammation, proliferation, and remodeling [71]. As shown in Table 6, based on the disc diffusion test, the three tested essential oils exhibited significant antibacterial activity against all tested bacteria compared to chloramphenicol (one-way ANOVA, *p ≤* 0.05). The highest inhibition zones were recorded by *Origanum vulgare,* followed by *Thymus vulgaris* and *Rosmarinus officinalis*. The results of the current study are in harmony with previously published reports that claimed the potent antimicrobial potential of *Origanum vulgare* [72], *Thymus vulgaris* [73], and *Rosmarinus officinalis* [74]. 

The minimum inhibitory concentration (MIC) and minimum bactericidal concentration (MBC) are represented in Table 7. The results supported the findings of the disc diffusion test, with low MIC and MBC values. Therefore, the most susceptible bacteria against *Origanum vulgare* were, in order, *S. aureus*, *L. monocytogenes*, *E. coli*, and *S. typhimurium*. For *Thymus vulgaris* essential oil, the most sensitive microorganisms were L. monocytogenes, *S. aureus*, *E. coli*, and *S. typhimurium*. In comparison, the most susceptible bacteria to *Rosmarinus officinalis* were, in order, L. monocytogenes, S. aureus, *E. coli*, and *S. typhimurium*. Indeed, the MIC test is required to evaluate the novel composite’s antibacterial properties traditionally used in wound healing [75]. In order to evaluate the nature of the antibacterial molecules, MBC/MIC values listed in Table 7 were calculated. All values were less than 4, meaning that these essential oils have bactericidal effects on the examined bacteria. It was published that when the MBC/MIC ratio is 4, the extract is regarded bactericidal, but when the MBC/MIC ratio is >4, it is deemed bacteriostatic [76]. Accordingly, based on the antibacterial evaluation of the tested essential oils, this composite is highly suggested for possible application for wound dressing and protection from wound infections.

### 3.5. Acute Dermal Irritation Assay

The skin irritation tests of 0.5% of the three essential oils did not show any severe type of irritation, and there was no evidence of any noticeable inflammation or redness observed. On the other hand, the 5% extract showed well-defined redness and moderate inflammation (Table 8). In summary, this study shows that low concentration (0.5%) of essential oils from either *Origanum vulgare*, *Thymus vulgaris*, or *Rosmarinus officinalis* had no toxic effects. Further, they did not elicit a hypersensitivity reaction or acute skin irritation at the low concentrations.

### 3.6. Wound Healing Study

The wound healing effect of *Thymus vulgaris* honey and its mixtures with *Origanum vulgare*, *Thymus vulgaris*, and *Rosmarinus officinalis* essential oils was observed in four groups of animals and compared with that of control (untreated) and standard (Madécassol cream 1%). The healing process was examined during the experimental period of 2 weeks for both thermal- and chemical-induced wounds to appraise the wound healing ability of the mixtures by following the wound closure. The results were then incorporated in Table 9.

Significant wound healing activity was shown in the groups treated by honey and mixtures compared to the reference group. 

**Figure 3:**
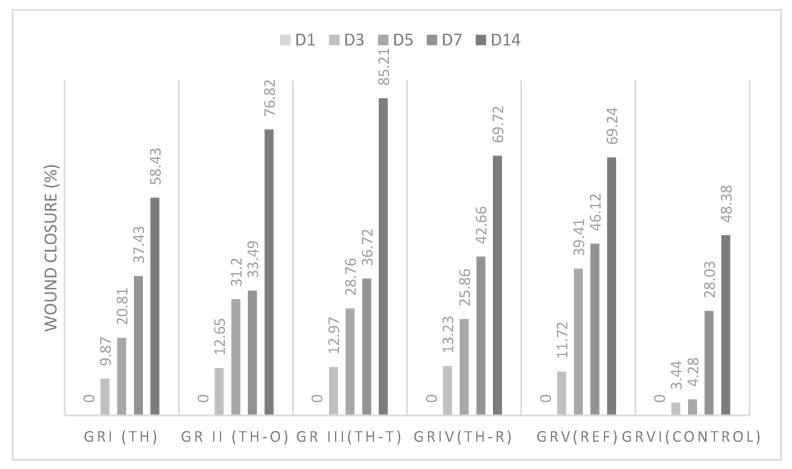
Wound healing effect of *Thymus vulgaris* honey and mixtures on thermal burns.

The wound healing activity in thermal-induced burns was found to be in the order TH-T > TH-O > TH-R > standard ≥ TH > control, as observed in Figure 3 during the first and second weeks of the test. The results indicated that the three mixtures were found to have a significant wound healing activity compared to that of the standard and the thyme honey alone. Among different mixtures, the mixture of *Thymus vulgaris* honey with *Thymus vulgaris* essential oil was found to possess the highest wound healing rate (85.21%) for thermally induced wounds at day 14.

**Figure 4:**
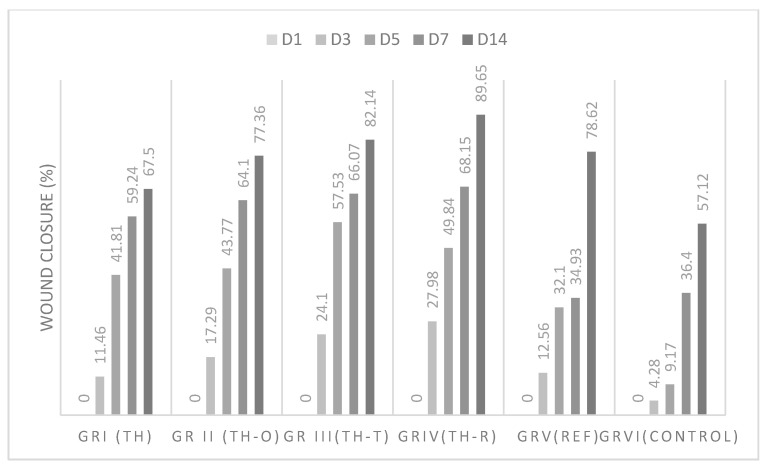
Wound healing effects of *Thymus vulgaris* honey and mixtures on chemical burns.

For chemical-induced wounds, the results are summarized in Table 9 and Figure 4, where the wound healing activity was found to be in the order of TH-R > TH-T > TH-O > TH > standard > control.

In both tests, all of the mixtures were found to have better wound healing activity than that of the honey alone and the positive control. Nevertheless, *Thymus vulgaris* essential oil combined with *Thymus vulgaris* honey showed outstanding wound healing activity, with wound closure rates of 85.21% and 82.14% in thermal- and chemical-induced burns, respectively, and expressed short healing time at days 5, 7, and 14.

The presence of thymol can justify the findings as the main component of the *Thymus vulgaris* essential oil [77]. This compound has been proven effective in boosting the wound care process in various studies; Buyana et al. showed the potential wound healing properties of topical gels loaded with thymol as dressings [78]. Pires et al. [79] also tested the addition of thymol to poly(dimethylsiloxane) on chitosan-alginate film. Since inflammation causes many difficulties, including infection, wound dehiscence, and impaired collagen synthesis, the anti-inflammatory effects of thymol would be promising material when thyme essential oil is used [60].

The remarkable qualities of *Thymus vulgaris* essential oil have been noted in comparison to other essential oils. According to the results of the comparative study of antimicrobial activities of essential oils of *Rosmarinus officinalis* and *Thymus vulgaris* carried out by Miladi et al. [80], *Thymus vulgaris* essential oil showed the higher bactericidal effect. In vitro, antioxidant and antihyperglycemic activities of thyme and oregano essential oils were compared. Thyme potently inhibited α-glucosidase (98.9%), against 50.5% for oregano [81].

The mixture of honey and essential oil of oregano has shown significant results. This effectiveness is consistent with the results reported in several studies [59,82,83,84,85]. The recent study by Avola et al. [86] also displayed the role of essential oil of oregano in wound re-epithelialization and re-establishment of skin remodeling.

The mixture of *Thymus vulgaris* honey and *Rosmarinus officinalis* essential oil has led to interesting results, with a wound closure rate of 89.65% in chemical-induced burns at day 14. Potential healing of rosemary essential oil was highlighted in the study of Abu-Al-Basal et al. [57], where *Rosmarinus officinalis* essential oil reduced inflammation and wound debridement when topically applied on wounds. The study of Abid et al. [87] also showed an obvious synergistic effect of hyaluronic acid, rosemary oil, and metronidazole to achieve better wound healing.

Even though conclusive results have been found, the exact mechanism of synergy between essential oils and honey is unclear, and no data have been found in the literature.

### 3.7. Chemical Composition Property of the Studied Essential Oils

The GC/MS analysis (Table 10) revealed that the primary chemical compounds detected in *Thymus vulgaris* essential oil were 1,3-cyclopentadiene (54.68%), g-terpinene (12.59%), p-cymene (11.11%), and thymol (9.23%), while the major chemical constituents of *Rosmarinus officinalis* essential oil were camphor (35.8%), eucalyptol (1,8-cineole) (26.22%), caryophyllene (14.25%), g-terpinene (7.77%) and α-terpinene (6.33%) and those for *Origanum vulgare* essential oil were ethanone (57.63%) and cyclopentadienes (19.79%) compounds, followed by g-terpinene (8.03%) and bicyclo[3.1.0]hex-2-ene (umbellulon) (7.24%) (Table 10). In harmony with our findings, the topical and systemic administration of thymol has shown remarkable anti-inflammatory activities in experimental models of edema and cutaneous inflammatory disorders [88], and the marked inhibitory effects of thymus oil therapy on wound-associated stasis, thrombosis, and tissue damage have been previously reported in burned wound models in rats [89]. Furthermore, the favored wound healing effects of oral and topical *T. vulgaris* EO therapy [60], or rosemary EO therapy [90], have been documented in surgically induced excision wound models in rats.

Likewise, the add-on topical camphor therapy with honey and sesame oil resulted in a significant acceleration in the healing, epithelization, and neovascularization in rats in experimental models of second-degree deep burn wound [91]. Cyclopentadienyl complexes [92] and ethanone derivatives [93] have also been explored as potent antimicrobial, antioxidant, and anticancer compounds via inhibition of reactive oxygen species and free radical production. Terpinenes (e.g., g- and α-terpinene and terpinen-4-ol) have also shown excellent antioxidant, analgesic, anti-inflammatory, anti-proliferative, and antimicrobial effects [94]. Most importantly, both terpinenes and 1,8-cineole (eucalyptol) have been recently recognized as promising therapeutic agents for accelerating wound healing and for suppressing microbial burden in skin injuries and dermatological disorders [95,96] via inhibition of the production of pro-inflammatory cytokines and other mediators in injured tissues [97].

As a further interest, both p-cymene and phellandrene have been described as among the main active components of topically applied essential oils for the treatment of wound modalities [98]. p-Cymene has also been shown to possess broad biological activity, including anti-inflammatory, analgesic, antioxidant, anti-inflammatory, anxiolytic, antimicrobial, and anticancer effects [99]. Despite this, further studies are still required to explore and identify the definite underlying mechanisms that could be beyond the favorable therapeutic effects of *Thymus vulgaris*, *Rosmarinus officinalis*, and *Origanum vulgare* essential oils in the wound healing process.

## 4. Conclusions

In this work, the wound healing activity of *Thymus vulgaris* honey from the region of Essaouira was evaluated and compared with mixtures of the same honey and three essential oils in order to enhance the synergy between these two natural components. The chemical and bacterial evaluation showed promising properties. The tests were performed in vivo on two models of burns on rabbits. For both types of burns, the mixtures were more effective in treating the wounds than honey alone or the positive control. However, the mixture that provided the best results was that of honey with *Thymus vulgaris* essential oil. Therefore, it may be used as a healing agent in different types of wounds in humans after certain clinical trials.

## Figures and Tables

**Table 1:** Plants used for wound healing in traditional Moroccan pharmacopoeia.

Information about the Herbalist	Ethnobotanical Characteristics Method of Preparation
Name Phone number Age Address (City) Locality Level of education Training Own use Ethnobotanical characteristics of the plants used for wound healing Local name Plant part used Form of use	Method of administration Effects felt Prescribers

**Table 2:** Questionnaire intended for beekeepers/honey used for wound healing in Morocco.

Information about the Beekeeper	Information about Honey
Name Phone number Age Address (City) Locality Level of education Own use Information about the apiary Size of the apiary Method of beekeeping Method of extraction Marketing circuit adapted	Most sold type(s) of honey Type(s) of honey used for wound healing Method of administration

**Table 3:** Sociodemographic characteristics and experience of beekeepers.

Characteristics	Number of Informants (*n*)	Frequency (%)
Age (years)		
<30	9	6.13
30–40	51	34.69
40–60	76	51.7
>60	11	7.48
Total	147	100
Gender		
Male	126	85.71
Female	21	14.29
Total	147	100
Education		
Illiterate	9	6.12
Primary/Koranic school	43	29.25
Secondary	71	48.3
University	24	16.33
Total	147	100
Apiary size		
<20	85	57.82
20 < A < 60	38	25.85
>60	24	16.33
Total	147	100

**Table 4:** Medicinal plants used in the treatment of wounds in Morocco as identified by the ethnopharmacological survey.

Latin Names	Local Names	Family	Part Used	Preparation	Type of Wound	Mode of Application	No. of Citations
*Acacia nilotica* L.	Sllaha	Fabaceae	Fruit	Poultice	Burns	Externally applied	3
*Ajuga iva* (L.) Schreb	Chendgura	Lamiaceae	Leaves	Decoction	Superficial wounds	Rinsing	2
*Aloe arborescens* Miller	Siber/Sabbar/Sabra	Aloeaceae	Leaves	Poultice	Burns	Externally applied	9
*Allium cepa* L.	Bessla	Amaryllidaceae	Bulb	Poultice	Burns	Externally applied	8
*Ammodaucus leucotrichus* Coss. & Durieu	Kmoun reg	Apiaceae	Seed	Powder	Wounds	Externally applied	2
*Argania Spinosa* (L.) Skeels	Argan	Sapotaceae	Seed	Oil	Skin regeneration/Skin care	Massage	4
*Artemisa herba-alba* Asso	Chih	Asteraceae	Leaves	Poultice	Wounds	Externally applied	8
*Calotropis procera* (Aiton.) W.T. Aiton	Tourja	Asclepiadaceae	Stem	Decoction	Wounds	Rinsing	6
*Cedrus atlantica* (Endl.) Manetti	Kdram	Pinaceae	Resin	Raw	Wounds	Externally applied	4
*Citrullus colocynthis* (L.) Schrad	Lhdej	Cucurbitacae	Fruit	Poultice	Wounds	Externally applied	2
*Euphorbia officinarum* subsp. Echinus (Hook. F. & Coss.)	Dghmouss	Euphorbiaceae	Stem	Powder	Wounds and abscesses	Externally applied	7
*Haloxylon scoparium* Pomel	Rremt	Amaranthaceae	Leaves	Poultice	Wounds	Externally applied	4
*Heliotropium curassavicum* L.	Lehbalia	Boraginaceae	Leaves	Powder	Wounds	Externally applied	1
*Launaea arborescens* (Batt.) Murb.	Oum bina	Asteraceae	Stem	Latex	Skin Care/Wounds	Massage	4
*Lavandula angustifolia* Mill.	Khouzama	Lamiaceae	Leaves	Essential oil/extract	Wounds/Burns/Skin care	Externally applied	5
*Lawsonia inermis* Linn.	Lhenna	Lythraceae	Leaves	Powder	Wounds/Burns/ Abscesses	Externally applied	7
*Marrubium vulgare* L.	Merewta	Lamiaceae	Leaves	Poultice	Wounds	Externally applied	4
*Nerium oleander* Linn.	Ddefla	Apocynaceae	Leaves	Powder	Burns	Externally applied	7
*Origanum vulgare* L.	Zaatar/setter	Lamiaceae	Leaves	Extract	Wounds/Burns	Externally applied	11
*Pinus pinaster* Aiton	Tayda	Pinaceae	Bark	Poultice	Wounds	Externally applied	5
*Ricinus communis* L.	Kharwae/Wriwra	Euphorbiaceae	Seeds	Oil	Wounds	Massage	3
*Rosmarinus officinalis* L.	Azir	Lamiaceae	Leaves	Powder	Burns/Wounds	Externally applied	14
*Salvia officinalis* L.	Ssalmiya	Lamiaceae	Leaves	Powder	Burns/Wounds	Externally applied	7
*Salvia verbenaca* L.	Khiyata	Lamiaceae	Leaves	Poultice	Wounds	Externally applied	4
*Senecio anteuphorbium* (L.) Sch.	Chbartou	Asteraceae	Stem	Poultice	Wounds	Externally applied	5
*Thymus satureioides* Coss.	Zaiitra/Tazukknit	Lamiaceae	Leaves	E.O./Extract	Burns/Wounds	Externally applied	7
*Thymus vulgaris* L.	Zaiitra, zaatar/āzukenni, tazukennit	Lamiaceae	Leaves	Extract	Wounds	Externally applied	11
*Thymus zygis* L.	Zaiitra/āzukenni, tazukennit	Lamiaceae	Leaves	E.O./Extract	Wounds	Externally applied	5
*Zygophyllum gaetulum* Emb. & Maire.	Laagaya	Zygophyllaceae	Stem	Powder	Wounds	Externally applied	3

**Table 5:** Physicochemical parameters of *Thymus vulgaris* honey.

Sample	Moisture (%)	pH	Free Acidity (Meq kg^−1^)	Lactonic Acidity (Meq kg^−1^)	Total Acidity (Meq kg^−1^)	Electrical Conductivity (ms cm^−1^)	Sugar Content (°Brix)	Ash (%)	HMF (mg/kg)
TH_1_	16.93	4.67	34.00	3.35	37.35	0.73	81.5	0.40	9.37
TH_2_	17.50	4.11	33.91	4.04	37.96	0.86	77.0	0.25	11.22
TH_3_	17.04	4.36	39.72	2.60	42.32	0.87	81.0	0.17	4.64
Mean	17.15	4.37	35.88	3.33	39.21	0.82	79.83	0.27	8.44
SD	0.30	0.26	3.32	0.72	2.71	0.083	2.46	0.12	3.43
Range	16.93–17.07	4.11–4.67	33.91–39.72	2.60–4.04	37.35–42.32	0.73–0.87	77–81.5	0.17–0.4	4.64–11.22
Codex	≤21%	3.4–6.1	≤50 meq/kg	_	8.68–59.49 meq/kg	≥0.700 (ms cm^−1^)	≥60 °Brix	≤0.6 g/kg	≤40 mg/kg

**Table 6:** Antibacterial activity of the essential oils of *Origanum vulgare, Thymus vulgaris*, and *Rosmarinus officinalis* compared to chloramphenicol (30 μg/disc).

Microorganisms	Mean Zone of Inhibition in Millimeters (Mean ± Standard Deviation) *			
	*Origanum Vulgare*	*Thymus Vulgaris*	*Rosmarinus Officinalis*	Chloramphenicol (30 μg)
*E. coli* ATCC 25922	25.1 ± 0.4	24.2 ± 0.3	17.2 ± 0.8	21.0 ± 0.4
*S. typhimurium* ATCC 700408	23.1 ± 0.6	20.3 ± 0.4	14.5 ± 0.6	12.6 ± 0.4
*S. aureus* ATCC 29213	30.4 ± 0.9	28.3 ± 1.1	21.8 ± 1.0	23.0 ± 0.6
*L. monocytogenes* ATCC 13932	34.4 ± 1.2	30.5 ± 0.7	24.9 ± 0.6	26.7 ± 0.9

* Mean of three replicates.

**Table 7:** MIC and MBC values of *Origanum vulgare, Thymus vulgaris*, and *Rosmarinus* officinalis essential oils *.

Microorganisms	*Origanum Vulgare*		*Thymus Vulgaris*		*Rosmarinus Officinalis*		Chloramphenicol	
	MIC mg/mL	MBC mg/mL	MIC mg/mL	MBC mg/mL	MIC mg/mL	MBC mg/mL	MIC μg/mL	MBC μg/mL
*E. coli* ATCC 25922	1.56	1.56	1.56	3.12	6.25	12.5	4	4
*S. typhimurium* ATCC 700408	3.12	3.12	3.12	6.25	12.5	25	64	64
*S. aureus* ATCC 29213	0.78	0.78	1.56	1.56	3.12	3.12	4	4
*L. monocytogenes* ATCC 13932	0.78	0.78	0.78	0.78	1.56	3.12	2	2

* MIC and MBC values of essential oils are interpreted in mg/mL, and the standard antibiotic is interpreted in μg/mL.

**Table 8:** Dermal observation in rats with *Origanum vulgare*, *Thymus vulgaris*, and *Rosmarinus officinalis* essential oils at 0.5% and 5% concentrations.

Rats	1 h		24 h		48 h		72 h		5 days	
	Erythema ^1^	Edema ^2^	Erythema	Edema	Erythema	Edema	Erythema	Edema	Erythema	Edema
GrII										
1	0	0	0	0	0	0	0	0	0	0
2	1	0	1	1	0	0	1	0	0	0
3	0	0	0	0	0	0	0	0	0	0
4	1	0	1	0	0	0	0	0	0	0
5	0	0	0	0	0	0	0	0	0	0
6	0	0	0	0	0	0	0	0	0	0
GrIII										
1	1	0	0	0	0	0	0	0	0	0
2	0	0	0	0	0	0	0	0	0	0
3	1	0	2	2	1	0	0	0	0	0
4	1	0	0	0	0	0	0	0	0	0
5	0	0	1	0	1	0	0	0	0	0
6	0	0	0	0	0	0	0	0	0	0
GrIV										
1	0	0	0	0	0	0	0	0	0	0
2	0	0	0	0	0	0	0	0	0	0
3	0	0	0	0	0	0	0	0	0	0
4	0	0	0	0	0	0	0	0	0	0
5	1	0	1	0	0	0	0	0	0	0
6	0	0	0	0	0	0	0	0	0	0
Gr V										
1	1	0	2	1	1	0	0	0	0	0
2	0	0	1	0	0	0	0	0	0	0
3	0	0	1	0	0	0	0	0	0	0
4	0	0	0	0	0	0	0	0	0	0
5	0	0	1	0	2	2	2	0	0	0
6	0	0	1	0	0	0	0	0	0	0
GrVI										
1	0	0	0	0	0	0	0	0	0	0
2	0	0	0	0	0	0	0	0	0	0
3	0	0	0	0	0	0	0	0	0	0
4	0	0	0	0	0	0	0	0	0	0
5	0	0	0	0	0	0	0	0	0	0
6	0	0	0	0	0	0	0	0	0	0
Gr VII										
1	1	1	1	1	0	0	0	0	0	0
2	1	0	2	2	1	0	0	0	0	0
3	1	0	2	1	2	2	2	2	1	0
4	0	0	0	0	0	0	0	0	0	0
5	0	0	0	0	0	0	0	0	0	0
6	0	0	0	0	0	0	0	0	0	0

^1^ Erythema was scored as follows: no erythema = 0, very slight erythema (barely perceptible) = 1, well-defined erythema = 2, moderate to severe erythema = 3, severe erythema (beet redness) to slight eschar formation (injuries in depth) = 4. ^2^ Edema formation was scored as follows: no edema = 1, very slight edema (barely perceptible) = 1, slight edema (edges of area well-defined by definite raising) = 2, moderate edema (raised approximately 1 mm) = 3, severe edema (raised more than 1 mm and extending beyond area of exposure).

**Table 9:** Effects of Thymus vulgaris honey and mixtures on wound contraction (%).

Rabbits	Day 1		Day 3		Day 5		1 Week		2 Weeks	
Groups	Thermal	Chemical	Thermal	Chemical	Thermal	Chemical	Thermal	Chemical	Thermal	Chemical
GrI (TH)	0.00	0.00	9.87 ± 0.14	11.46 ± 0.21	20.81 ± 0.68	41.81 ± 1.13	37.43 ± 0.12	59.24 ± 1.76	58.43 ± 1.45	67.5 ± 0.42
Gr II (TH-O)	0.00	0.00	12.65 ± 0.68	17.29 ± 1.14	31.2 ± 1.17	43.77 ± 0.74	33.49 ± 0.35	64.1 ± 0.66	76.82 ± 0.78	77.36 ± 1.12
Gr III(TH-T)	0.00	0.00	12.97 ± 1.17	24.1 ± 0.45	28.76 ± 1.49	57.53 ± 1.06	36.72 ± 1.14	66.07 ± 1.12	85.21 ± 1.36	82.14 ± 0.41
GrIV (TH-R)	0.00	0.00	13.23 ± 1.13	27.98 ± 0.3	25.86 ± 0.84	49.84 ± 0.79	42.66 ± 1.71	68.15 ± 0.72	69.72 ± 0.7	89.65 ± 1.03
GrV (Ref)	0.00	0.00	11.72 ± 0.31	12.56 ± 0.73	39.41 ± 1.13	32.1 ± 0.84	46.12 ± 0.38	34.93 ± 0.21	69.24 ± 1.19	78.62 ± 0.2
GrVI (Control)	0.00	0.00	3.44 ± 1.92	4.28 ± 0.49	7.93 ± 0.45	9.17 ± 1.96	28.03 ± 0.13	36.4 ± 1.58	48.38 ± 0.27	57.12 ± 0.86

Values are given as mean ± standard deviation for groups of six rats each.

**Table 10:** Chemical composition of the studied essential oils.

NO	TEO ^a^		REO ^b^		OEO ^c^	
	Compound	%	Compound	%	Compound	%
1	1,3-Cyclopentadiene	54.68	Camphor	35.8	Ethanone	57.63
2	γ-Terpinene	12.59	Eucalyptol	26.22	Ethyl-tetramethyl-cyclopentadiene	11.64
3	p-Cymene	11.11	Caryophyllene	14.25	1,3-Cyclopentadiene	8.15
4	Thymol	9.23	γ-Terpinene	7.77	γ-Terpinene	8.03
5	Phenol	4.69	α-Terpinene	6.33	Umbellulon (Bicyclol [3.1.0]Hex-2-ene)	7.24
6	Caryophyllene	2.77	Bicycloheptan-2-ol	3.34	Trichloromethane	3.00
7	2-Carene	1.39	Dodecane	1.92	2-Carene	1.04
8	Linalool	1.38	Terpinen-4-ol	1.83	Linalool	1.01
9	β-Myrcene	1.26	Humulene	1.62	p-Cymene	0.68
10	Umbellulon (Bicyclol [3.1.0] Hex-2-ene)	0.99	4-Carene	0.50	β-Phellandrene	0.48
11	1,2-Propanediol	0.73	Pinocarvone	0.40	β-Myrcene	0.29

^a^ Thymus vulgaris essential oil; ^b^ Rosmarinus officinalis essential oil; ^c^ Origanum vulgare essential oil.

## Data Availability

The data presented in this study are available on request from the corresponding author.
